# The Relationship between the Lunar Phase, Menstrual Cycle Onset and Subjective Sleep Quality among Women of Reproductive Age

**DOI:** 10.3390/ijerph18063245

**Published:** 2021-03-21

**Authors:** Yoko Komada, Makoto Sato, Yuko Ikeda, Azusa Kami, Chika Masuda, Shigenobu Shibata

**Affiliations:** 1 Faculty of Liberal Arts, Meiji Pharmaceutical University, Tokyo 204-8588, Japan; 2 MTI Ltd., Tokyo 163-1435, Japan; sato_ma@mti.co.jp (M.S.); azazpion@gmail.com (A.K.); chika20424@gmail.com (C.M.); 3 Laboratory of Physiology and Pharmacology, School of Advanced Science and Engineering, Waseda University, Tokyo 162-0056, Japan; yuko.ikeda_cd02@fuji.waseda.jp (Y.I.); shibatas@waseda.jp (S.S.)

**Keywords:** sleep quality, menstruation, menstrual cycle, lunar rhythms, moon, reproductive health, human

## Abstract

The aim of the present study was to investigate the association among lunar cycle, menstrual cycle onset, and subjective sleep quality. Menstrual cycle onset data from the six most recent menstrual cycles were obtained for 529 women (aged 25–39 years) using the smartphone app Luna Luna. We also collected questionnaire survey data on sleep quality from each participant. Overall, there was no association between the onset of menstrual cycle and lunar phase. Interestingly, the proportion of good sleepers with menstrual cycle beginning during the light period was significantly higher than that during the dark period, while the proportion of poor sleepers with menstrual cycle beginning during the dark period was significantly higher than that during the light period. When participants were categorized by the combination of lunar phases (light, dark, neutral periods) in the two most recent menstrual cycle onsets, the “both dark period” group and the “other (light and dark) period” group showed the lowest proportion of good sleepers. Menstrual cycle onset in the dark period was associated with a deterioration in subsequent subjective sleep quality, which was more apparent with consecutive onsets in the dark period or at a rapidly changing lunar phase.

## 1. Introduction

Organisms have evolved to live with geophysical cycles of different period lengths. Adaptations of animals to the lunar cycle (approximately 29.5 days) are frequently observed. The reproductive cycles of marine organisms are linked to changing levels of moonlight and the tidal cycle, both of which are governed by the phases of the moon [1,2]. Published data suggest that organisms such as palolo worms, which are polychaete worms living on coral islands, have an endogenous circalunar clock [3]. Furthermore, other animals are clearly influenced by the moon and possess internal clocks that can predict the lunar cycle [4]. Moonlight has been revealed to control the regular oscillation of clock gene expression in fish [5], suggesting that the light/dark periods of the lunar cycle affect the daily expression of clock genes and influence the sleep and/or diurnal rhythms of biochemical reactions.

The effects of the lunar phase on the human reproductive rhythm have not yet been elucidated, although mature women have a 29.5-day menstrual cycle [6]. It has been hypothesized that there are lunar effects on human biology including psychosis, violent behavior, birth, and menstruation, although most of these proposed influences of the moon on human physiology have not been able to be statistically confirmed [4]. The question of whether lunar changes might somehow entrain the menstrual rhythm has been discussed for many years. A report published in 1806 by the French physician J. A. Murat concluded that the human menstrual cycle is not governed by the lunar cycle [7]. However, the idea that the lunar cycle was responsible for regulation of the menstrual cycle regained interest by the end of the twentieth century [7]. A study using four separate datasets gathered in different years and seasons demonstrated that women tend to menstruate during a full moon with a diminishing likelihood of the onset of menstrual cycle with increasing time from full moon [8]. Recently, long-term menstrual recordings of individual women with distinct methods for biological rhythm analysis suggested that menstrual cycles were intermittently synchronous with the luminescence and/or gravimetric cycles of the moon [9].

The factors associated with the human menstrual cycle are age [10,11], body mass index (BMI) [10,12], disrupted circadian rhythms [13], and psychological distress [14]. A database of more than 120,000 women indicated that the mean menstrual cycle length was 29.9 ± 5.5 days in 25–29 year old women and decreased with increasing age with a mean difference of 2.9 days between the youngest (18–24 years) and oldest (40–45 years) cohorts [10]. The mean variation in cycle length was 0.4 days or 14% higher in women with a BMI of over 35 relative to women with a BMI of 18.5–25 [10]. Female shift-workers are more likely to report menstrual irregularity and longer menstrual cycles compared to non-shift-workers, indicating disrupted circadian rhythms [13]. Young women with more than 1 h of social jetlag, the discrepancy between sleep times on workdays and those on work-free days, had more severe menstrual symptoms than those with less than 1 h of social jetlag [15]. In addition, the menstrual cycle has effects on sleep; poorer sleep quality in the premenstrual phase and menstruation is common in women [16]. Thus, it has been suggested that there are significant relationships between reproductive health and sleep among women [17].

One potential mechanism underlying the link between psychological distress and menstrual problems is the influence of the hypothalamic–pituitary–adrenal (HPA) axis on gonadotropin releasing hormone (GnRH). Increased reactivity of the HPA axis induced by psychological distress subsequently delays or impedes the luteinizing hormone (LH) surge [18,19]. Therefore, investigations of human menstrual cycles must consider these associated factors. In addition, the association between menstrual cycle onset in the light/dark period and sleep quality has not been elucidated, although it is known that the light period of the lunar cycle influences human sleep structure [20,21].

In this study, we aimed to investigate the relationship between lunar phase and the menstrual cycle onset date obtained from a mobile phone app. In addition, we examined the association among lunar cycle, menstrual cycle onset among women of reproductive age, and subjective sleep quality. It is hypothesized that the relationship between the menstrual cycle initiation date and lunar phase differs according to age, sleep quality, and physical and mental health. Therefore, we conducted a questionnaire survey of participants about their subjective sleep quality and physical and mental health.

## 2. Materials and Methods

### 2.1. Participants

All study procedures were conducted in accordance with the guidelines outlined in the Declaration of Helsinki. The Ethical Review Board of Waseda University, Japan approved the study protocol (No. 2017-156). The details of the study and explanation of its aims were explained to the participants on the web, and their online informed consent was obtained.

The present study used data obtained via a mobile phone app from a commercial women’s health care service, Luna Luna (MTI Ltd., Tokyo, Japan). Luna Luna is a total health care service for female mobile phone users. Each user records the dates when they recognized menstrual bleeding (onset of menstruation). Luna Luna offers its users predictions of menstrual cycles, fertility, ovulation, and related health care information, based on user-entered personal records that are sent to, and stored in, its data server. The data are securely stored separately from personally identifiable information. Luna Luna has operated as a commercial service since 2000, with a total of 14 million downloads [22].

**Figure 1:**
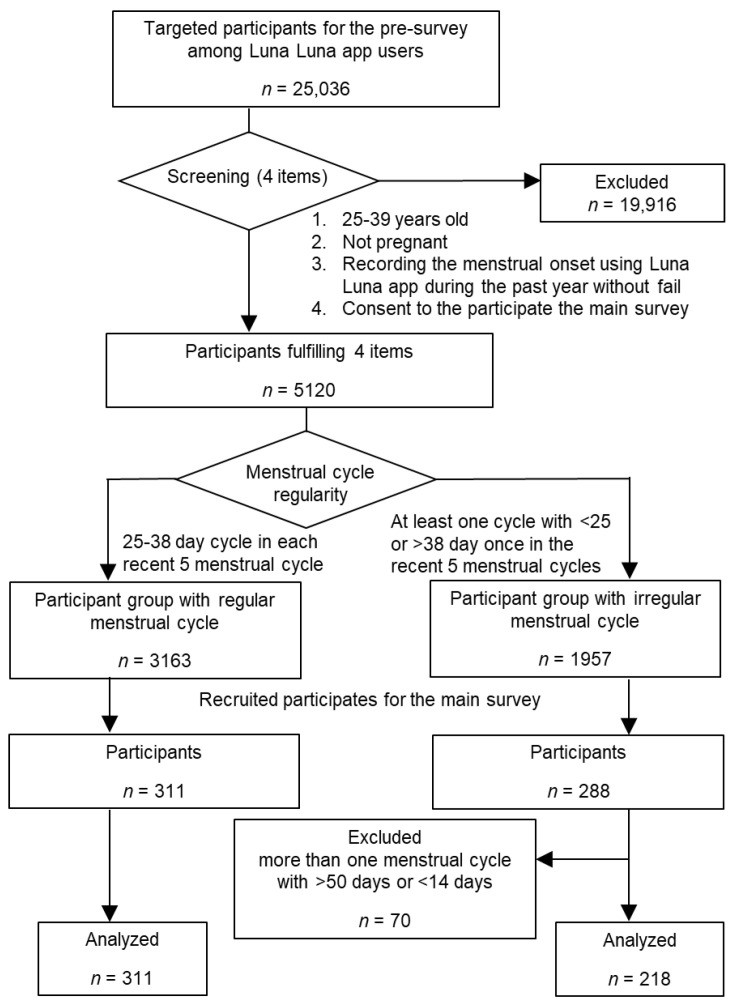
Study design flowchart.

A flowchart of the study design is shown in Figure 1. We published a notice of the research goals regarding “menstrual cycle and chronotype” on the Luna Luna app and asked participants to answer a screening survey. This survey comprised four items requesting the following information: (1) age, (2) the frequency with which they recorded their menstrual cycle onset on the Luna Luna app during the past year, (3) whether they were pregnant or not, and (4) whether they would consent to participating in the main survey requiring approximately 20 min. There were 5120 women fulfilling the following criteria: (1) 25–39 years old, (2) not pregnant, (3) menstrual cycle onset was recorded on the Luna Luna app during the past year without fail, and (4) consented to participate in the main survey. We analyzed the five most recent menstrual cycles of these participants and classified them into two groups: regular menstrual cycle, with each cycle lasting 25–38 days (*n* = 3163); and irregular menstrual cycle, with at least one cycle of <25 or >38 days (*n* = 1957). We recruited participants for the main survey via the Luna Luna app, with the intention of enrolling 300 participants in each group. A $5 gift card was offered to the participants of the main survey. We were able to enroll 311 and 288 participants in the regular and irregular menstrual cycle groups, respectively. Of these participants, 70 women recorded more than one cycle with >50 days or <14 days and these data were excluded from our analysis to eliminate the possibility of participant input error (Figure 1).

### 2.2. Materials

With consent from each participant, their six most recent menstrual cycle onset dates—accounting for the lengths of the five most recent menstrual cycles—were obtained from the Luna Luna data server. All participants also filled out the following questionnaires about menstrual symptoms, sleep quality and sleep habits, quality of life, and demographic variables.

### 2.3. Menstrual Symptoms

The participants’ experience of menstrual symptoms within the previous few months was reported. A modified 35-item Japanese version of the Menstrual Distress Questionnaire (mMDQ) was used to assess the proportion of subjects experiencing menstrual pain and symptom severity. The questionnaire includes the following subscales: pain, concentration, behavioral change, autonomic reactions, water retention, and negative effects [23]. Item response options consist of a 4-point scale ranging from no experience of the symptom to an acute or partially disabling experience of the symptom during the menstrual period compared with the post-menstrual period, which is defined as the few days after menstruation. Higher scores indicate severer symptoms. Cronbach’s alpha for the total score for the scale was 0.95.

### 2.4. Sleep Quality and Sleep Habits

The Pittsburgh Sleep Quality Index (PSQI) is the most common measure of subjective sleep quality over the last month [24,25]. The first four items enquire about the participant’s bedtime, number of minutes it took for them to fall asleep, awakening time, and hours of sleep per night. In a recent modification of the original PSQI, Pilz et al. replaced “usual” with explicit references to sleep on workdays or work-free days [26]. In this study, we asked separate questions about sleep behavior on workdays and work-free days. Social jetlag was calculated as the absolute difference between mid-sleep on work-free days and mid-sleep on workdays [27]. The next items ask how often the participants had trouble sleeping for various reasons (e.g., woke up in the middle of the night, needed to go to the bathroom, was coughing, bad dreams). Each item is scored on a 4-point scale ranging from “never” to “three times a week or more”. Additional items include a subjective rating of sleep quality (scored on a 4-point scale from 0 = “very good” to 3 = “very bad”), the use of sleep medication (scored on a 4-point scale ranging from 0 = “never” to 3 = “three or more times a week”), and trouble staying awake during the day (scored on a 4-point scale ranging from 0 = “never” to 3 = “three or more times a week”). The final item measures motivation and enthusiasm to get things done (scored on a 4-point scale ranging from 0 = “no problem at all” to 3 = “a very big problem”). The total score ranges from 0 to 21, with a higher score representing poorer sleep quality. The cut-off score for sleep disturbance has been established at 5.5 points. The psychometric properties of both the English and the Japanese version of the PSQI are good [24,25].

### 2.5. Quality of Life

The standardized 8-item Short-Form Health Survey of the Medical Outcomes Study was used to assess health-related quality of life [28]. The survey comprises 8 questions allocated to two summary scores, namely, physical health component summary score (PCS) and mental health component summary score (MCS), with higher scores indicating better health. PCS and MCS were calculated using a norm-based scoring method based on the national standard values determined from large-scale survey data for the general Japanese population (the mean score and standard deviation for the general Japanese population are 50 and 10, respectively). Cronbach’s alpha for the scale was 0.82.

### 2.6. Demographic Variables

Sociodemographic and lifestyle characteristics of participants were obtained, that is, age, height, weight, smoking status (yes/no), alcohol consumption (yes/no), job (full-time worker/part-time worker/student/housewife), shift work (yes/no), duration of screen time involving electronic media such as smartphone after 8 pm, and number and age of children.

### 2.7. Analyses

An almanac was used to determine when the new moon/full moon had occurred. The lunar cycle was categorized into the light period and the dark period according to the method of Cutler et al. [29] The light period was defined as the period from the midpoint between the new moon and the full moon (the first quarter) to the midpoint between the full moon and new moon (the last quarter). The dark period was defined as the period from the midpoint between the full moon and the new moon (the last quarter) to the midpoint between the new moon and the full moon (the first quarter). For example, a new moon occurred on 26 May 2017 at 4:44, a full moon on 9 June 2017 at 22:10, a new moon on 24 June 2017 at 11:31, and a full moon on 9 July 2017 at 13:07. The midpoint between the new moon and the full moon was 2 June 2017 13:27, the midpoint between the full moon and the new moon was 17 June 2017 4:50, and the midpoint between the new moon and the full moon was 2 July 2017 0:19. Therefore, the dark period was from 3 June 2017 to 16 June 2017 (14 days), and the light period was from 17 June 2017 to 1 July 2017 (15 days). In addition to the above categories, the lunar cycle was divided into the light period around the full moon (7 days), the dark period around the new moon (7 days), and the neutral period (15.5 days) to provide a clear distinction between the light and dark periods.

The number of subjects reporting menstrual cycle onset during the light and dark periods were calculated based on the lunar phase. Thereafter, the numbers of subjects with menstrual cycle onset in the two periods were compared using the binominal test. Participants were categorized into good sleepers (PSQI < 5.5) or poor sleepers (PSQI ≥ 5.5) [24,25], and the proportions of the six most recent menstrual cycle start dates in the light and dark periods were compared using a chi-squared test. Statistical analysis was performed using SPSS version 26 (IBM SPSS, Armonk, NY, USA), and BellCurve (Social Survey Research Information Co., Ltd, Tokyo, Japan). The level of significance was set at *p* < 0.05.

## 3. Results

### 3.1. Comparison of Menstrual Cycle Onset Data between the Light and Dark Periods

For these analyses, the lunar cycle was categorized into the light period and dark period according to the method of Cutler et al. [29].

The number of subjects who reported menstrual cycle onset was counted for each day of the lunar cycle, and these numbers were compared between the light and dark periods (the period of analysis was 26 May 2017–21 August 2017). We noted 768 and 791 menstrual cycle onsets during the light and dark periods, respectively, and the counts did not differ significantly (*z* = −0.58, *p* = 0.56).

### 3.2. Comparison of Menstrual Cycle Onset Data between the Light and Dark Periods by Age and BMI

To test whether the relationship between menstrual cycle onset and lunar phase differs by age, the numbers of menstrual cycle onsets in the light and dark periods were compared among the different age groups (25–29 years old, 30–34 years old, and 35–39 years old). There were no significant differences in the proportion of participants with the menstrual cycle onset in the light and dark periods in the 25–29 age group (*n* = 192, light period: 259 (47%), dark period: 293 (53%), *z* = −1.45, *p* = 0.15), in the 30–34 age group (*n* = 189, light period: 294 (53%), dark period: 265 (47%), *z* = 1.23, *p* = 0.22), and in the 35–39 age group (*n* = 148, light period: 215 (48%), dark period: 233 (52%), *z* = −0.85, *p* = 0.40).

Comparison of the proportions of participants with menstrual cycle onset in the light and dark periods showed no significant differences in the higher BMI group (BMI ≥ 20.5, *n* = 260, light period: 386 (50%), dark period: 383 (50%), z = 0.11, *p* = 0.91) or the lower BMI group (BMI < 20.5, *n* = 269, light period: 382 (48%), dark period: 408 (52%), z = −0.93, *p* = 0.36).

### 3.3. Comparison of Menstrual Cycle Onset Data between the Light and Dark Periods in Good and Poor Sleepers

The numbers of menstrual cycle onsets during the light and dark periods were compared between good sleepers and poor sleepers, as categorized by the Pittsburgh Sleep Quality Index (PSQI) [24,25]. The numbers of menstrual cycle onsets during the light and dark periods were respectively 345 (55%) and 287 (45%) among good sleepers, and 423 (46%) and 504 (54%) among poor sleepers. The binominal test revealed that the proportion of good sleepers with menstrual cycle onset during the light period was significantly higher than that during the dark period (z = 2.31, *p* = 0.02), while the proportion of poor sleepers with menstrual cycle onset during the dark period was significantly higher than that during the light period (z = −2.66, *p* = 0.01).

Comparisons between good and poor sleepers showed no significant differences in mean age (good sleepers: 31.6 years old, poor sleepers: 31.5 years old, *t*(527) = 0.20, *p* = 0.85), BMI (good sleepers: 21.1, poor sleepers: 21.6, *t*(527) = −0.84, *p* = 0.40), or menstrual cycle length (good sleepers: 29.9 days, poor sleepers: 30.2 days, *t*(527) = −1.66, *p* = 0.10). Physical health component summary (PCS), mental health component summary (MCS) [28], and menstrual symptom (the modified Menstrual Distress Questionnaire (mMDQ)) [23] scores were significantly worse among poor sleepers than among good sleepers (PCS: 50.4 vs. 47.8, *t*(527) = 4.39, *p* < 0.001; MCS: 46.1 vs. 40.8, *t*(527) = 7.65, *p* < 0.001; mMDQ: 27.4 vs. 38.3, *t*(527) = −6.69, *p* < 0.001).

In addition, we categorized the PCS, MCS, and mMDQ scores into high and low groups and compared the number of menstrual cycle onsets during the light and dark periods. The binominal test revealed that the proportion of menstrual cycle onset was significantly higher during the dark period than in the light period among the low-PCS group (z = −2.11, *p* = 0.04), whereas there were no significant differences in the proportion of menstrual cycle onsets between the light period and dark period among the high-PCS group (z = 1.29, *p* = 0.20), the high- and low-MCS groups (z = 0.14, *p* = 0.89; z = −0.96, *p* = 0.34, respectively), and the high- and low-mMDQ groups (z = 0.04, *p* = 0.97; z = −0.87, *p* = 0.39, respectively).

### 3.4. Association of Subjective Sleep Quality with the Lunar Phase of Recent Menstrual Cycle Onsets

**Figure 2:**
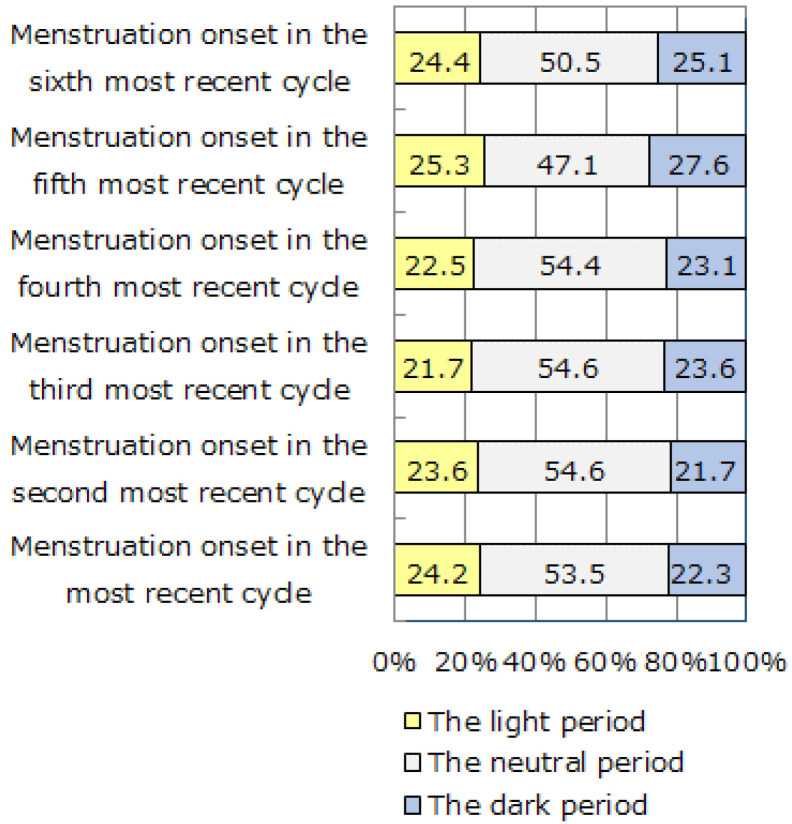
The proportions of participants with menstruation onset in the light, dark, and neutral periods in their six most recent menstrual cycles (*n* = 529). There was no significant difference in the proportions of participants with menstrual cycle onset in the light and dark periods during each of the six menstrual cycles.

For this analysis, the lunar cycle was divided into the light period around the full moon (7 days), the dark period around the new moon (7 days), and the neutral period (15.5 days), in order to provide a clear distinction between the light and dark periods. The dates of initiation of the six most recent menstrual cycles were categorized into the light period, the dark period, and the neutral period. Figure 2 indicates the proportions of participants with their menstrual cycle onset in the light period, the dark period, and the neutral period in each cycle. There was no significant difference in the proportions of participants with menstrual cycle onset in the light and dark periods during each of the six menstrual cycles (*χ*^2^(5) = 0.44, *p* = 0.99).

**Figure 3:**
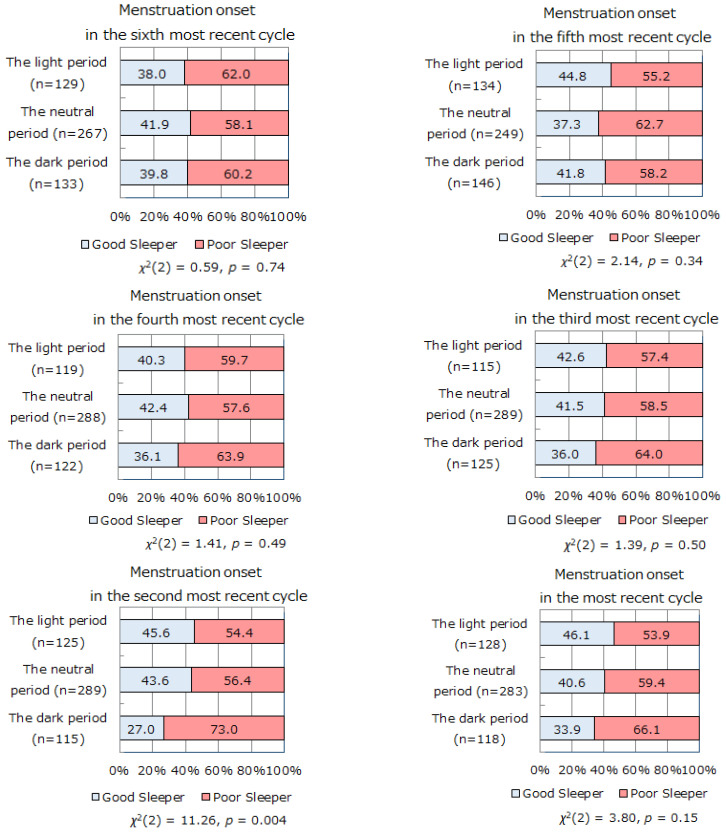
The proportions of good sleepers and poor sleepers with menstruation onset in the light/dark/neutral periods during their six most recent menstrual cycles. The proportion of subjective poor sleepers was significantly higher in participants with menstrual cycle onset in the dark period than in those with onset in the light period, in the second most recent menstrual cycle. The same tendency was observed in the most recent menstrual cycle.

Next, we investigated the relationship between lunar phase at menstrual cycle onset and subjective sleep quality. Figure 3 shows the proportions of good and poor sleepers at the time of the survey (over a 1-month time interval) in the categories of light, dark, and neutral periods of the lunar cycle during the six most recent menstrual cycles. In the second most recent menstrual cycle, the proportion of subjective poor sleepers was significantly higher in participants with menstrual cycle onset in the dark period than in those with the onset in the light period (*χ*^2^(2) = 11.26, *p* = 0.004). The same tendency was observed in the most recent menstrual cycle, although it did not reach statistical significance (*χ*^2^(2) = 3.80, *p* = 0.15).

**Figure 4:**
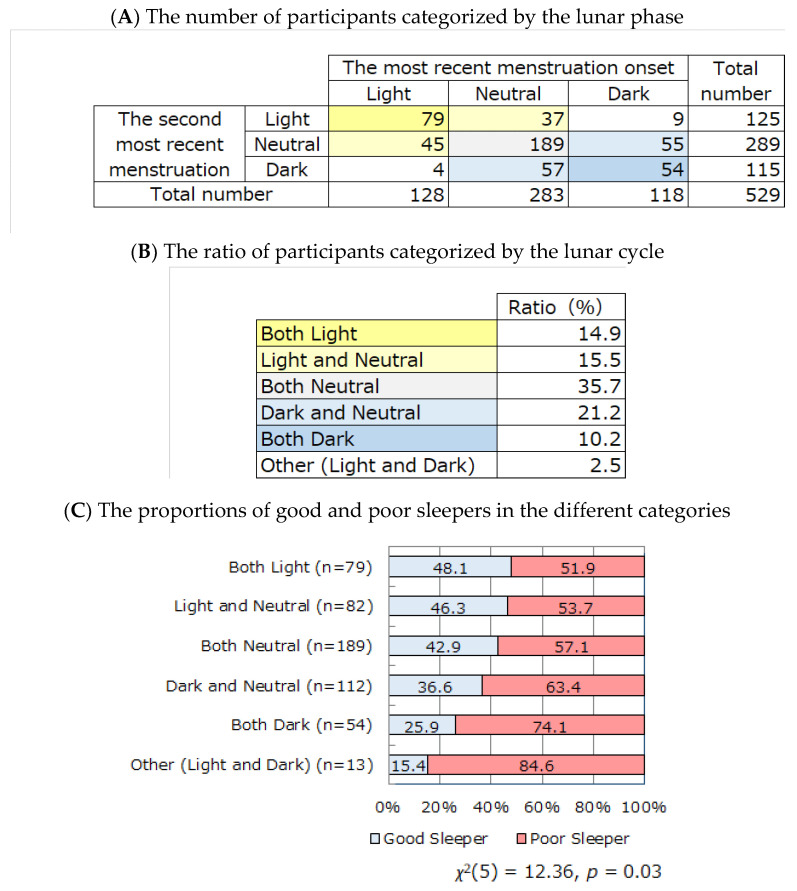
The relationship between subjective sleep quality and the combination of lunar phase (light, dark, neutral periods) in the two most recent menstruation onsets. Participants were categorized by the combination of lunar phases at the most recent and the second most recent onset of menstrual cycle (**A**,**B**), and the proportions of good and poor sleepers were calculated (**C**). The proportion of good sleepers was highest in the “both light period” group, followed by the “light and neutral period” group and the “both neutral period” group. The “both dark period” group and the “other (light and dark period)” group showed the lowest proportions of good sleepers.

Assuming that lunar phase (the light or dark period) at the menstrual cycle onset during the second most recent menstrual cycle potentially affects subjective sleep quality over a 1-month time interval, we hypothesized that the same lunar phase during the two most recent menstrual cycle onsets (e.g., the light period at the most recent menstrual cycle onset and the light period at the second most recent menstrual cycle onset, or the dark period at the most recent menstrual cycle onset and the dark period at the second most recent menstrual cycle onset) would enhance the influence of lunar phase on sleep quality. Therefore, participants were categorized by the combination of lunar phases at the most recent and the second most recent onset of menstrual cycle (Figure 4A,B), and the proportions of good and poor sleepers were calculated (Figure 4C). A significant difference was observed in the proportions of good and poor sleepers among the groups (*χ*^2^(5) = 12.36, *p* = 0.03). The proportion of good sleepers was highest in the “both light period” group, followed by the “light and neutral period” group and the “both neutral period” group. The “both dark period” group and the “other (light and dark period)” group showed the lowest proportions of good sleepers.

**Figure 5:**
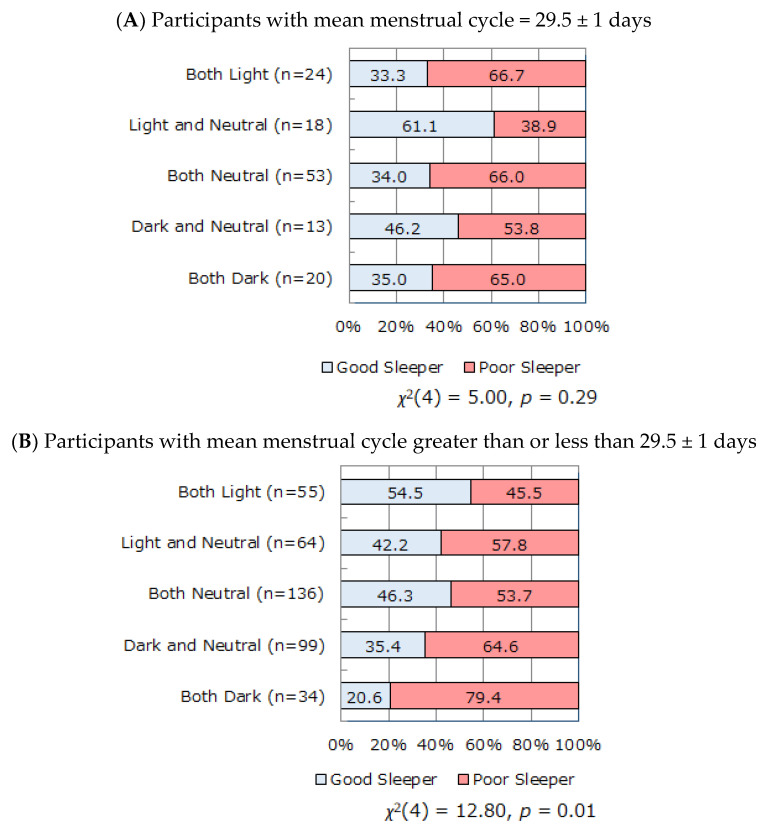
The relationship between the combination of lunar phase (light, dark, neutral periods) in the two most recent menstruation onsets and subjective sleep quality among participants with mean menstrual cycle = 29.5 ± 1 days (**A**) and those with mean menstrual cycle greater than or less than 29.5 ± 1 days (**B**). There was no statistically significant relationship between lunar phase and sleep quality among participants with a menstrual cycle of 29.5 ± 1 days (**A**). In contrast, there was a significant relationship between lunar phase and sleep quality, with a gradual change in lunar phase at the onset of menstrual cycle due to longer or shorter mean menstrual cycle length (**B**).

The relationship between the lunar phase in the two most recent menstrual cycle onsets and the proportions of good and poor sleepers was explored separately among the participants whose mean menstrual cycle corresponded to the lunar cycle (29.5 ± 1 days) and among those whose mean menstrual cycle was longer or shorter than 29.5 days (Figure 5A,B). When the menstrual cycle is 29.5 days, menstruation starts at the same lunar phase as the previous menstruation. When the menstrual cycle is not 29.5 days, the lunar phase at menstrual cycle onset changes gradually. We conducted separate analyses of participants with menstrual cycles equal to 29.5 ± 1 days and those with menstrual cycles greater than or less than 29.5 ± 1 days to reveal any influence of either lunar phase or change of lunar phase on sleep. With a gradual change in lunar phase at the onset of menstrual cycle due to longer or shorter mean menstrual cycle length (Figure 5B), there was a significant relationship between sleep quality and lunar phase of the two most recent menstrual cycle onsets (*χ*^2^(4) = 12.80, *p* = 0.01). In contrast, there was no statistically significant relationship between lunar phase and sleep quality among participants with a menstrual cycle of 29.5 ± 1 days (*χ*^2^(4) = 5.00, *p* = 0.29, Figure 5A).

## 4. Discussion

This study aimed to elucidate the relationship between menstrual cycle onset and lunar phase. We extracted menstrual cycle onset data from the six most recent menstrual cycles for 529 women (25–39 years old) using the Luna Luna smartphone app. We also collected data on self-reported sleep quality, menstrual symptoms, and quality of life from the participants. The lunar phase (i.e., light and dark periods) at the beginning of menstrual cycles was analyzed in detail. The numbers of menstrual cycle onsets in the light and dark periods were nearly equal overall, suggesting that lunar phase did not influence menstrual cycle onset. In addition, there was no significant association between lunar phase and menstrual cycle onset when analyzing this relationship by age or BMI. Notably, the proportion of good sleepers with menstrual cycle beginning during the light period was significantly higher than that during the dark period, while the proportion of poor sleepers with menstrual cycle beginning during the dark period was significantly higher than that during the light period.

It is clear that some animals are influenced by the moon and have internal clocks that can predict the lunar cycle, and that human culture has been affected by the phases of the moon [4]. However, there has yet to be convincing evidence that the moon can affect the biology of humans [4,30]. From a report published in the early nineteenth century to a recent review paper, it has been suggested that the human menstrual cycle is not governed by the lunar phase [7]. However, despite the lack of statistical evidence, beliefs remain strong that more babies are born during a full moon instead of new moon, and that aspects of human reproductive health, such as deliveries and menstruation, are associated with lunar phase [4]. A previous study using four separate datasets gathered in different years and seasons demonstrated that women tend to menstruate during a full moon, with a diminishing likelihood of the onset of menstrual cycle with increasing time from full moon [8]. In contrast, another study of 826 female volunteers aged 16–25 with a normal menstrual cycle found that a large proportion of menstruations occurred around the new moon [31].

To the best of our knowledge, there has been no previous study investigating the relationships among menstrual cycle onset, lunar phase, and subjective sleep quality. The present study revealed that the proportion of good sleepers with menstrual cycle onset during the light period was significantly higher than that during the dark period, while the proportion of poor sleepers with menstrual cycle beginning during the dark period was significantly higher than that during the light period. This prompts the question: does menstruation during the dark period lead to sleep quality deterioration, or do poor sleepers tend to have a menstrual cycle onset during the dark period? To investigate this, we analyzed subjective sleep quality and the lunar phase of the onset of menstrual cycle over a 1-month time interval since the six most recent menstruation cycles. The proportion of poor sleepers evaluated by self-reported questionnaire (PSQI ≥ 5.5) was significantly higher when the two most recent menstrual cycle occurred in the dark period than that when they occurred in the light or neutral periods (Figure 3). We can infer that self-reported sleep quality is associated with the lunar phase of the two most recent menstrual cycle onsets. The occurrence of the two most recent menstrual cycles in the same lunar phase (light/dark period) seemed to enhance the influence on sleep quality. The proportion of good sleepers was highest in the “both light period” group, followed by the “light and neutral period” group and the “both neutral period” group. The “both dark period” group and the “other (light and dark period)” group showed the lowest proportion of good sleepers (Figure 4). Our findings suggest that the occurrence of menstrual cycle in the dark period leads to sleep quality deterioration.

To investigate whether the lunar phase or change of lunar phase have a stronger influence on subjective sleep evaluation, separate analyses were conducted among the participants whose mean menstrual cycle corresponded to the lunar cycle (29.5 ± 1 days) and among those whose mean menstrual cycle was longer or shorter than 29.5 days. The former participants showed no significant relationship between lunar phase and sleep quality. The latter participants, for whom the lunar phase at the onset of menstrual cycle changed gradually from cycle to cycle, showed a significant relationship between sleep quality and the lunar phase of the two most recent onsets of menstrual cycle (Figure 5). We speculate that subjective sleep quality is vulnerable to the lunar phase (dark period) at menstrual cycle onset among participants whose menstrual cycles are out of lunar cycle, whereas lunar phase has less influence on sleep among participants whose menstrual cycle coincides with the lunar cycle. Furthermore, menstrual cycle onset in the dark period was associated with a deterioration in subjective sleep quality, in particular for consecutive onsets in the dark period or at a rapidly changing phase (light to dark period, dark to light period). The underlying mechanism of this phenomenon is unclear.

One possibility is the interaction of melatonin, a hormonal marker of the circadian timing system. Cajochen et al. revealed that evening melatonin level was significantly lower around the full moon compared to that at other lunar phases [20]. The disharmony of kinetics in the secretion of melatonin and estrogen, specifically the enhancement of estrogen during the light period in which melatonin secretion is diminished, can deteriorate sleep quality over the following one to two months. The three-way interaction between melatonin fluctuation, the secretion of hormones that show circalunar rhythms (e.g., estrogen, progesterone, gonadotropic hormone, and GnRH), and lunar cycle would affect subjective sleep quality among women. A recent paper reported that sleep cycles in people oscillate during the 29.5-day lunar cycle: sleep starts later and is shorter on the nights before the full moon, when moonlight is available during the hours following dusk [32]. It is thus necessary to comprehensively consider the inclusion of changes in melatonin receptor sensing.

Another possibility is the effect of repetitive moonlight illumination during the light period on the expression of clock genes [5], which would have a good influence on sleep. Recent research investigating the relationship between human birth and lunar cycle revealed that lunar phase did not influence the total number of births [33]. However, further detailed analyses on the subset of infants categorized by time of birth revealed that childbirths occurred more frequently during the nighttime hours at or around full moon compared to the other lunar phases. In contrast, the childbirths during the daytime hours were more frequent at or around the daytime manifestation of the new moon compared to the other lunar phases [33]. A strictly controlled experiment revealed that cortical activity and human sleep were synchronized with lunar phases [34]. Lunar rhythms are not as evident as circadian rhythms, and are thus difficult to interpret. Unravelling the mechanism underlying circalunar rhythms in humans remains challenging.

This study has some limitations. First, data were drawn from a mobile phone app used in Japan. The sample size was not large enough, and we did not obtain the ethnic origin and sociodemographic data of the participants. Therefore, interpretation and generalization of results should be done cautiously. Second, sleep quality was collected by retrospective self-report, which has a potential for recall bias. Objective measures of sleep duration and some sleep problems, such as actigraphy, would be desirable.

## 5. Conclusions

Our findings highlight the association among menstrual cycle onset, lunar phase, and subjective sleep quality. Overall, there was no association between the menstrual cycle onset and lunar phase. However, menstrual cycle onset in the dark period was associated with a deterioration in subsequent subjective sleep quality, which was more apparent with consecutive onsets in the dark period or at a rapidly changing lunar phase.
